# *N*-(4-Bromo­phen­yl)-2-(naphthalen-1-yl)acetamide

**DOI:** 10.1107/S1600536811041110

**Published:** 2011-10-12

**Authors:** Hoong-Kun Fun, Ching Kheng Quah, B. Narayana, Prakash S. Nayak, B. K. Sarojini

**Affiliations:** a X-ray Crystallography Unit, School of Physics, Universiti Sains Malaysia, 11800 USM, Penang, Malaysia; b Department of Studies in Chemistry, Mangalore University, Mangalagangotri 574 199, Mangalore, India; c Department of Chemistry, P. A. College of Engineering, Nadupadavu, Montepadavu, PO, Mangalore 574 153, India

## Abstract

In the title compound, C_18_H_14_BrNO, the naphthalene ring system and the benzene ring form dihedral angles of 78.8 (2) and 19.7 (2)°, respectively, with the acetamide C—C(=O)—N plane. The naphthalene ring system forms a dihedral angle of 64.88 (19)° with the benzene ring. In the crystal, mol­ecules are linked *via* inter­molecular bifurcated (N,C)—H⋯O hydrogen bonds, generating an *R*_2_^1^(6) ring motif, forming chains along the *b* axis.

## Related literature

For the structural similarity of *N*-substituted 2-aryl­acetamides to the lateral chain of natural benzyl­penicillin, see: Mijin & Marinkovic (2006 ▶); Mijin *et al.* (2008 ▶). For the coordination abilities of amides, see: Wu *et al.* (2008 ▶, 2010 ▶). For studies of amides in therapy, myocardial infarction and ischemic disease, see: Dorsch *et al.* (2002 ▶); Wang, Li & Li (2010 ▶); Wang, Beck *et al.* (2010 ▶). For related structures, see: Fun *et al.* (2010 ▶); Li & Wu (2010 ▶); Xiao *et al.* (2010 ▶); Praveen *et al.* (2011 ▶). For standard bond-length data, see: Allen *et al.* (1987 ▶). For the definition of graph-set notation, see: Bernstein *et al.* (1995 ▶).
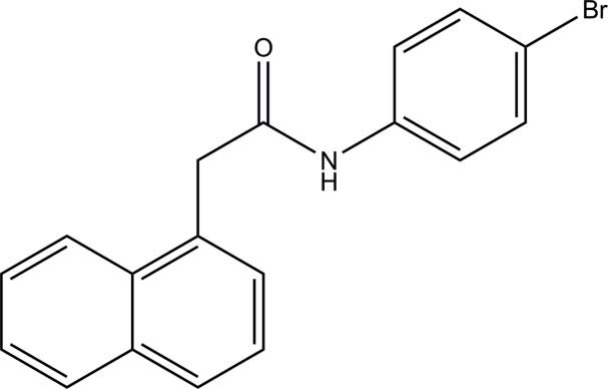


## Experimental

### 

#### Crystal data

C_18_H_14_BrNO
*M* *_r_* = 340.21
Orthorhombic, 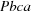

*a* = 12.6837 (11) Å
*b* = 9.4047 (11) Å
*c* = 25.641 (3) Å
*V* = 3058.6 (6) Å^3^
*Z* = 8
Mo *K*α radiation
μ = 2.69 mm^−1^
*T* = 296 K
0.40 × 0.30 × 0.28 mm

#### Data collection

Bruker SMART APEXII DUO CCD area-detector diffractometer
Absorption correction: multi-scan (*SADABS*; Bruker, 2009 ▶) *T*_min_ = 0.415, *T*_max_ = 0.523
18691 measured reflections
2999 independent reflections
1864 reflections with *I* > 2σ(*I*)
*R*_int_ = 0.046

#### Refinement

*R*[*F* ^2^ > 2σ(*F* ^2^)] = 0.051
*wR*(*F* ^2^) = 0.182
*S* = 1.02
2999 reflections
184 parameters
H-atom parameters constrained
Δρ_max_ = 0.37 e Å^−3^
Δρ_min_ = −0.53 e Å^−3^

### 

Data collection: *APEX2* (Bruker, 2009 ▶); cell refinement: *SAINT* (Bruker, 2009 ▶); data reduction: *SAINT*; program(s) used to solve structure: *SHELXTL* (Sheldrick, 2008 ▶); program(s) used to refine structure: *SHELXTL*; molecular graphics: *SHELXTL*; software used to prepare material for publication: *SHELXTL* and *PLATON* (Spek, 2009 ▶).

## Supplementary Material

Crystal structure: contains datablock(s) global, I. DOI: 10.1107/S1600536811041110/is2786sup1.cif

Structure factors: contains datablock(s) I. DOI: 10.1107/S1600536811041110/is2786Isup2.hkl

Supplementary material file. DOI: 10.1107/S1600536811041110/is2786Isup3.cml

Additional supplementary materials:  crystallographic information; 3D view; checkCIF report

## Figures and Tables

**Table 1:** Hydrogen-bond geometry (Å, °)

*D*—H⋯*A*	*D*—H	H⋯*A*	*D*⋯*A*	*D*—H⋯*A*
N1—H1*N*1⋯O1^i^	0.80	2.09	2.879 (3)	167
C11—H11*A*⋯O1^i^	0.97	2.59	3.422 (4)	143

Symmetry code: (i) 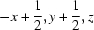.

**Fig. 1.**
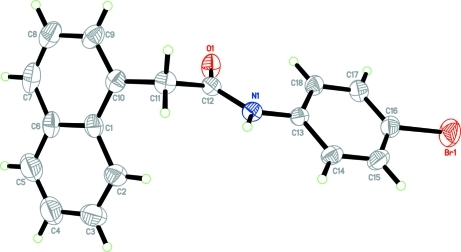
The molecular structure of the title compound, showing 20% probability displacement ellipsoids for non-H atoms.

**Fig. 2.**
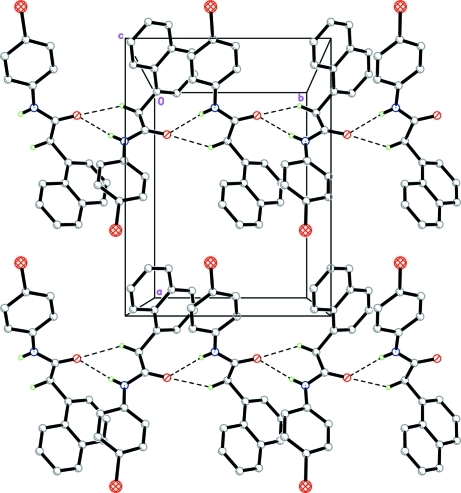
The crystal structure of the title compound, viewed along the c axis. H atoms not involved in hydrogen bonds (dashed lines) have been omitted for clarity.

## supplementary crystallographic information

### Comment

*N*-Substituted 2-arylacetamides are very interesting compounds because of their structural similarity to the lateral chain of natural benzylpenicillin (Mijin & Marinkovic, 2006; Mijin *et al.*, 2008). Amides are also used as ligands due to their excellent coordination abilities (Wu *et al.*, 2008, 2010) and for the therapy of thromboembolic disorder and effective anticoagulants for myocardial infarction and ischemic disease (Dorsch *et al.*, 2002; Wang, Li & Li, 2010; Wang, Beck *et al.*, 2010). Crystal structures of some acetamide derivatives, viz., 2-(4-bromophenyl)-*N*-(2-methoxyphenyl)acetamide (Xiao *et al.*, 2010), *N*-benzyl-2-(2-bromophenyl)-2-(2-nitrophenoxy) acetamide (Li & Wu, 2010) and *N*-(3-chloro-4-fluorophenyl)-2- (naphthalen-1-yl)acetamide (Praveen *et al.*, 2011) have been reported. In view of the importance of amides, we report herein the crystal structure of the title compound.

The molecular structure is shown in Fig. 1. Bond lengths (Allen *et al.*, 1987) and angles are within normal ranges and are comparable to a related structure (Fun *et al.*, 2010). The naphthalene ring system (C1–C10, maximum deviation of 0.022 (7) Å at atom C7) and the benzene ring (C13–C18) form dihedral angles of 78.8 (2) and 19.7 (2)°, respectively, with the acetamide moiety [O1/N1/C11/C12, maximum deviation of 0.014 (4) Å at atom C12]. The naphthalene ring system also forms dihedral angle of 64.88 (19)° with the benzene ring.

In the crystal packing (Fig. 2), molecules are linked *via* intermolecular bifurcated N1—H1N1···O1 and C11—H11A···O1 acceptor bonds (Table 1), generating an R_2_^1^(6) ring motif, (Bernstein *et al.*, 1995) to form one-dimensional chains along the [010] direction.

### Experimental

Naphthalen-1-acetic acid (0.186g, 1 mmol) and 4-bromoaniline (0.172g, 1 mmol) were dissolved in dichloromethane (20 ml). The mixture was stirred in presence of triethylamine at 273 K for about 3 h. The contents were poured into 100 ml of ice-cold aqueous hydrochloric acid with stirring, and was extracted thrice with dichloromethane. Organic layer was washed with saturated NaHCO_3_ solution and brine solution, dried and concentrated under reduced pressure to give the title compound. Single crystals were grown from toluene and acetone mixture by the slow evaporation method (*m.p.*: 476-478 K).

### Refinement

Atom H1N1 was located from the difference Fourier map and refined using a riding model, with N1—H1N1 = 0.80 Å, and with *U*_iso_(H) = 1.2*U*_eq_(N). The remaining H atoms were positioned geometrically and refined using a riding model, with C—H = 0.93 or 0.97 Å and *U*_iso_(H) = 1.2 *U*_eq_(C). The same *U*_ij_ parameters were used for atom pair C4/C5.

### Crystal data

**Table:**

C_18_H_14_BrNO	*F*(000) = 1376
*M**_r_* = 340.21	*D*_x_ = 1.478 Mg m^−^^3^
Orthorhombic, *P**b**c**a*	Mo *K*α radiation, λ = 0.71073 Å
Hall symbol: -P 2ac 2ab	Cell parameters from 3596 reflections
*a* = 12.6837 (11) Å	θ = 2.3–21.0°
*b* = 9.4047 (11) Å	µ = 2.69 mm^−^^1^
*c* = 25.641 (3) Å	*T* = 296 K
*V* = 3058.6 (6) Å^3^	Block, colourless
*Z* = 8	0.40 × 0.30 × 0.28 mm

### Data collection

**Table:**

Bruker SMART APEXII DUO CCD area-detector diffractometer	2999 independent reflections
Radiation source: fine-focus sealed tube	1864 reflections with *I* > 2σ(*I*)
graphite	*R*_int_ = 0.046
φ and ω scans	θ_max_ = 26.0°, θ_min_ = 1.6°
Absorption correction: multi-scan (*SADABS*; Bruker, 2009)	*h* = −15→15
*T*_min_ = 0.415, *T*_max_ = 0.523	*k* = −11→11
18691 measured reflections	*l* = −29→31

### Refinement

**Table:**

Refinement on *F*^2^	Primary atom site location: structure-invariant direct methods
Least-squares matrix: full	Secondary atom site location: difference Fourier map
*R*[*F*^2^ > 2σ(*F*^2^)] = 0.051	Hydrogen site location: inferred from neighbouring sites
*wR*(*F*^2^) = 0.182	H-atom parameters constrained
*S* = 1.02	*w* = 1/[σ^2^(*F*_o_^2^) + (0.081*P*)^2^ + 1.7994*P*] where *P* = (*F*_o_^2^ + 2*F*_c_^2^)/3
2999 reflections	(Δ/σ)_max_ = 0.001
184 parameters	Δρ_max_ = 0.37 e Å^−^^3^
0 restraints	Δρ_min_ = −0.53 e Å^−^^3^

### Special details

**Table:**

Geometry. All esds (except the esd in the dihedral angle between two l.s. planes) are estimated using the full covariance matrix. The cell esds are taken into account individually in the estimation of esds in distances, angles and torsion angles; correlations between esds in cell parameters are only used when they are defined by crystal symmetry. An approximate (isotropic) treatment of cell esds is used for estimating esds involving l.s. planes.
Refinement. Refinement of F^2^ against ALL reflections. The weighted R-factor wR and goodness of fit S are based on F^2^, conventional R-factors R are based on F, with F set to zero for negative F^2^. The threshold expression of F^2^ > 2sigma(F^2^) is used only for calculating R-factors(gt) etc. and is not relevant to the choice of reflections for refinement. R-factors based on F^2^ are statistically about twice as large as those based on F, and R- factors based on ALL data will be even larger.

### Fractional atomic coordinates and isotropic or equivalent isotropic displacement parameters (Å^2^)

**Table:**

	*x*	*y*	*z*	*U*_iso_*/*U*_eq_	
Br1	0.68647 (5)	0.59626 (9)	0.26162 (3)	0.1354 (4)	
O1	0.2893 (2)	0.3465 (2)	0.09635 (13)	0.0798 (8)	
N1	0.3185 (2)	0.5799 (3)	0.11054 (13)	0.0606 (7)	
H1N1	0.2943	0.6557	0.1026	0.073*	
C1	0.0129 (3)	0.4200 (4)	0.07382 (18)	0.0776 (11)	
C2	0.0061 (4)	0.5306 (5)	0.11333 (19)	0.0917 (13)	
H2A	0.0608	0.5956	0.1175	0.110*	
C3	−0.0795 (5)	0.5381 (7)	0.1437 (2)	0.1183 (18)	
H3A	−0.0837	0.6095	0.1687	0.142*	
C4	−0.1616 (5)	0.4428 (8)	0.1390 (3)	0.1343 (16)	
H4A	−0.2200	0.4522	0.1606	0.161*	
C5	−0.1592 (5)	0.3370 (9)	0.1039 (3)	0.1343 (16)	
H5A	−0.2151	0.2734	0.1015	0.161*	
C6	−0.0718 (3)	0.3231 (5)	0.07082 (19)	0.0889 (13)	
C7	−0.0643 (5)	0.2134 (6)	0.0329 (3)	0.1152 (19)	
H7A	−0.1179	0.1463	0.0306	0.138*	
C8	0.0194 (6)	0.2039 (6)	−0.0004 (2)	0.1104 (17)	
H8A	0.0219	0.1337	−0.0260	0.133*	
C9	0.1015 (4)	0.3026 (4)	0.00486 (18)	0.0880 (12)	
H9A	0.1595	0.2944	−0.0171	0.106*	
C10	0.1007 (3)	0.4098 (4)	0.04051 (16)	0.0719 (10)	
C11	0.1911 (3)	0.5111 (4)	0.04556 (17)	0.0749 (10)	
H11A	0.1636	0.6049	0.0534	0.090*	
H11B	0.2272	0.5167	0.0123	0.090*	
C12	0.2695 (3)	0.4708 (3)	0.08686 (15)	0.0604 (8)	
C13	0.4021 (3)	0.5751 (3)	0.14681 (13)	0.0573 (8)	
C14	0.4212 (3)	0.6974 (4)	0.17541 (16)	0.0740 (10)	
H14A	0.3773	0.7759	0.1716	0.089*	
C15	0.5052 (4)	0.7029 (5)	0.20955 (17)	0.0906 (13)	
H15A	0.5180	0.7851	0.2287	0.109*	
C16	0.5701 (3)	0.5868 (5)	0.21535 (18)	0.0838 (12)	
C17	0.5511 (3)	0.4651 (5)	0.18744 (18)	0.0819 (11)	
H17A	0.5950	0.3866	0.1915	0.098*	
C18	0.4673 (3)	0.4587 (4)	0.15334 (16)	0.0707 (10)	
H18A	0.4545	0.3758	0.1346	0.085*	

### Atomic displacement parameters (Å^2^)

**Table:**

	*U*^11^	*U*^22^	*U*^33^	*U*^12^	*U*^13^	*U*^23^
Br1	0.1139 (5)	0.1942 (8)	0.0981 (5)	−0.0291 (4)	−0.0306 (3)	−0.0162 (4)
O1	0.0801 (16)	0.0393 (12)	0.120 (2)	−0.0026 (10)	−0.0274 (15)	0.0069 (13)
N1	0.0672 (16)	0.0380 (12)	0.077 (2)	−0.0013 (11)	0.0057 (14)	0.0018 (13)
C1	0.069 (2)	0.082 (3)	0.082 (3)	0.0096 (19)	−0.017 (2)	0.011 (2)
C2	0.089 (3)	0.090 (3)	0.096 (3)	0.015 (2)	−0.002 (3)	−0.005 (3)
C3	0.112 (4)	0.123 (4)	0.120 (4)	0.025 (3)	0.013 (3)	−0.014 (4)
C4	0.087 (2)	0.174 (5)	0.143 (4)	−0.001 (3)	−0.005 (3)	0.002 (3)
C5	0.087 (2)	0.174 (5)	0.143 (4)	−0.001 (3)	−0.005 (3)	0.002 (3)
C6	0.079 (3)	0.088 (3)	0.100 (3)	−0.006 (2)	−0.035 (2)	0.013 (3)
C7	0.110 (4)	0.099 (4)	0.137 (5)	−0.015 (3)	−0.061 (4)	0.005 (4)
C8	0.136 (5)	0.088 (3)	0.107 (4)	0.014 (3)	−0.048 (4)	−0.014 (3)
C9	0.113 (3)	0.070 (2)	0.081 (3)	0.011 (2)	−0.027 (2)	−0.005 (2)
C10	0.082 (2)	0.064 (2)	0.070 (2)	0.0090 (18)	−0.018 (2)	0.013 (2)
C11	0.085 (2)	0.057 (2)	0.082 (3)	−0.0006 (17)	−0.005 (2)	0.011 (2)
C12	0.0600 (18)	0.0450 (17)	0.076 (2)	−0.0011 (14)	0.0071 (16)	0.0031 (17)
C13	0.0647 (18)	0.0478 (16)	0.059 (2)	−0.0103 (14)	0.0104 (16)	0.0009 (15)
C14	0.090 (3)	0.061 (2)	0.071 (2)	−0.0100 (18)	0.014 (2)	−0.0094 (19)
C15	0.108 (3)	0.091 (3)	0.073 (3)	−0.027 (3)	0.013 (2)	−0.024 (2)
C16	0.078 (3)	0.106 (3)	0.068 (3)	−0.019 (2)	0.002 (2)	−0.003 (2)
C17	0.072 (2)	0.081 (2)	0.093 (3)	−0.0039 (19)	−0.003 (2)	0.003 (2)
C18	0.072 (2)	0.0562 (18)	0.084 (3)	−0.0029 (16)	−0.0043 (19)	−0.0047 (19)

### Geometric parameters (Å, °)

**Table:**

Br1—C16	1.896 (4)	C8—C9	1.402 (8)
O1—C12	1.220 (4)	C8—H8A	0.9300
N1—C12	1.345 (4)	C9—C10	1.361 (6)
N1—C13	1.410 (5)	C9—H9A	0.9300
N1—H1N1	0.8031	C10—C11	1.496 (5)
C1—C10	1.407 (6)	C11—C12	1.501 (5)
C1—C6	1.411 (6)	C11—H11A	0.9700
C1—C2	1.455 (6)	C11—H11B	0.9700
C2—C3	1.337 (7)	C13—C18	1.382 (5)
C2—H2A	0.9300	C13—C14	1.385 (5)
C3—C4	1.379 (8)	C14—C15	1.380 (6)
C3—H3A	0.9300	C14—H14A	0.9300
C4—C5	1.342 (10)	C15—C16	1.374 (7)
C4—H4A	0.9300	C15—H15A	0.9300
C5—C6	1.401 (8)	C16—C17	1.371 (6)
C5—H5A	0.9300	C17—C18	1.378 (6)
C6—C7	1.421 (8)	C17—H17A	0.9300
C7—C8	1.366 (8)	C18—H18A	0.9300
C7—H7A	0.9300		
			
C12—N1—C13	128.4 (3)	C9—C10—C1	117.7 (4)
C12—N1—H1N1	112.7	C9—C10—C11	121.6 (4)
C13—N1—H1N1	119.0	C1—C10—C11	120.7 (4)
C10—C1—C6	121.7 (4)	C10—C11—C12	114.1 (3)
C10—C1—C2	121.2 (4)	C10—C11—H11A	108.7
C6—C1—C2	117.1 (4)	C12—C11—H11A	108.7
C3—C2—C1	119.4 (5)	C10—C11—H11B	108.7
C3—C2—H2A	120.3	C12—C11—H11B	108.7
C1—C2—H2A	120.3	H11A—C11—H11B	107.6
C2—C3—C4	121.9 (6)	O1—C12—N1	123.1 (3)
C2—C3—H3A	119.1	O1—C12—C11	121.3 (3)
C4—C3—H3A	119.1	N1—C12—C11	115.6 (3)
C5—C4—C3	121.5 (7)	C18—C13—C14	119.2 (4)
C5—C4—H4A	119.2	C18—C13—N1	123.7 (3)
C3—C4—H4A	119.2	C14—C13—N1	117.0 (3)
C4—C5—C6	119.5 (6)	C15—C14—C13	120.1 (4)
C4—C5—H5A	120.2	C15—C14—H14A	119.9
C6—C5—H5A	120.2	C13—C14—H14A	119.9
C5—C6—C1	120.6 (5)	C16—C15—C14	120.1 (4)
C5—C6—C7	122.4 (5)	C16—C15—H15A	120.0
C1—C6—C7	117.1 (5)	C14—C15—H15A	120.0
C8—C7—C6	121.9 (5)	C17—C16—C15	120.1 (4)
C8—C7—H7A	119.1	C17—C16—Br1	120.1 (4)
C6—C7—H7A	119.1	C15—C16—Br1	119.8 (3)
C7—C8—C9	118.3 (5)	C16—C17—C18	120.2 (4)
C7—C8—H8A	120.9	C16—C17—H17A	119.9
C9—C8—H8A	120.9	C18—C17—H17A	119.9
C10—C9—C8	123.4 (6)	C17—C18—C13	120.3 (4)
C10—C9—H9A	118.3	C17—C18—H18A	119.9
C8—C9—H9A	118.3	C13—C18—H18A	119.9
			
C10—C1—C2—C3	178.9 (4)	C2—C1—C10—C11	1.3 (5)
C6—C1—C2—C3	−1.7 (6)	C9—C10—C11—C12	−95.0 (4)
C1—C2—C3—C4	0.6 (8)	C1—C10—C11—C12	83.0 (4)
C2—C3—C4—C5	0.6 (11)	C13—N1—C12—O1	3.8 (6)
C3—C4—C5—C6	−0.5 (11)	C13—N1—C12—C11	−173.5 (3)
C4—C5—C6—C1	−0.6 (9)	C10—C11—C12—O1	34.1 (5)
C4—C5—C6—C7	180.0 (6)	C10—C11—C12—N1	−148.6 (3)
C10—C1—C6—C5	−178.9 (5)	C12—N1—C13—C18	18.9 (5)
C2—C1—C6—C5	1.7 (6)	C12—N1—C13—C14	−164.0 (3)
C10—C1—C6—C7	0.5 (6)	C18—C13—C14—C15	0.7 (5)
C2—C1—C6—C7	−178.9 (4)	N1—C13—C14—C15	−176.5 (3)
C5—C6—C7—C8	177.6 (5)	C13—C14—C15—C16	−0.1 (6)
C1—C6—C7—C8	−1.9 (7)	C14—C15—C16—C17	−0.3 (6)
C6—C7—C8—C9	2.5 (8)	C14—C15—C16—Br1	179.1 (3)
C7—C8—C9—C10	−1.9 (7)	C15—C16—C17—C18	0.2 (7)
C8—C9—C10—C1	0.6 (6)	Br1—C16—C17—C18	−179.2 (3)
C8—C9—C10—C11	178.7 (4)	C16—C17—C18—C13	0.4 (6)
C6—C1—C10—C9	0.0 (5)	C14—C13—C18—C17	−0.8 (6)
C2—C1—C10—C9	179.4 (4)	N1—C13—C18—C17	176.2 (4)
C6—C1—C10—C11	−178.0 (3)		

### Hydrogen-bond geometry (Å, °)

**Table:**

*D*—H···*A*	*D*—H	H···*A*	*D*···*A*	*D*—H···*A*
N1—H1N1···O1^i^	0.80	2.09	2.879 (3)	167
C11—H11A···O1^i^	0.97	2.59	3.422 (4)	143

Symmetry codes: (i) −*x*+1/2, *y*+1/2, *z*.
